# Postoperative Flare and Corneal Endothelial Cell Loss After Eight-Chop Technique Phacoemulsification: A Prospective Observational Study

**DOI:** 10.3390/jcm15020557

**Published:** 2026-01-09

**Authors:** Tsuyoshi Sato

**Affiliations:** Department of Ophthalmology, Sato Eye Clinic, Nemoto 3-3, Matsudo-shi 271-0077, Chiba-ken, Japan; perfect-eightchop@sato-ganka.com

**Keywords:** aqueous flare, cataract surgery, corneal endothelial cell, eight-chop technique, phacoemulsification

## Abstract

**Objectives**: The Eight-chop technique is a mechanically based nuclear segmentation method designed to improve surgical efficiency and reduce intraocular tissue stress during phacoemulsification. Early postoperative aqueous flare serves as an objective indicator of surgical invasiveness, whereas corneal endothelial cell density (CECD) loss represents a structural measure of endothelial injury. Although both parameters are clinically important, their relationship has not been systematically investigated in the context of this newer mechanical fragmentation approach. **Methods**: This prospective observational study included 118 eyes from 70 non-diabetic patients undergoing uncomplicated Eight-chop phacoemulsification. Aqueous flare was measured preoperatively and at postoperative Day 1, Day 7, Week 7, and Week 19 using laser flare photometry. CECD was evaluated preoperatively and at Weeks 7 and 19. Changes over time were analyzed using paired *t*-tests. Linear mixed-effects models (random intercept = patient ID) were constructed to assess predictors of CECD loss and postoperative intraocular pressure (IOP) reduction. Explanatory variables included Day 1 flare, age, preoperative CECD, nucleus hardness (Emery-Little grade), cumulative dissipated energy (CDE), and irrigation fluid volume. **Results**: Postoperative flare increased significantly at all time points (all *p* < 0.001), peaking on Day 7 (16.7 ± 9.21 photon counts/ms). CECD loss was extremely small, averaging 1.38% at Week 7 and 1.46% at Week 19. In mixed-effects models, Day 1 flare was not associated with CECD loss at Week 7 (*p* = 0.35) or Week 19 (*p* = 0.85). Significant predictors of CECD loss included Emery-Little grade (*p* = 0.004 at Week 7; *p* = 0.025 at Week 19), with borderline contributions from CDE and irrigation volume. IOP decreased significantly at Weeks 7 and 19; however, Day 1 flare did not predict IOP reduction. **Conclusions**: Eight-chop phacoemulsification produced uniformly low postoperative inflammation and exceptionally small corneal endothelial cell loss. Early postoperative flare did not predict CECD loss, suggesting that the Eight-chop technique provides a highly standardized, low-invasiveness surgical environment. These findings suggest that the Eight-chop technique lowers ultrasound energy requirements and may help reduce corneal endothelial stress relative to standard phacoemulsification.

## 1. Introduction

Cataract surgery is the most frequently performed ophthalmic surgical procedure worldwide and provides substantial improvement in visual function [1]. Since the introduction of phacoemulsification in 1967 [2], continuous refinements in surgical techniques have been made, leading to improved operative efficiency and reduced surgical invasiveness. Various nucleus fragmentation techniques have been developed, including the divide-and-conquer technique [3,4,5], Chip-and-Flip [6], phaco-chop [4,5], Stop-and-Chop [7], Quick-Chop [8], and the Phaco Pre-chop technique [9]. Among them, Akahoshi’s Phaco Pre-chop technique mechanically divides the nucleus before phacoemulsification, achieving marked reductions in cumulative ultrasound energy, aspiration time, and irrigating fluid volume [9]. However, because of its technical difficulty, it has not gained widespread adoption [10]. Eight-chop, developed as a modification of the Phaco Pre-chop technique, was introduced in 2023 as a novel method of nuclear fragmentation [11].

Among parameters reflecting surgical invasiveness, postoperative aqueous flare and loss of corneal endothelial cell density (CECD) are clinically meaningful indicators of postoperative inflammation and corneal endothelial injury, respectively [12]. Numerous studies have evaluated CECD loss following divide-and-conquer and phaco-chop techniques [13,14,15], whereas studies focusing on postoperative flare are relatively limited [12]. Furthermore, very few investigations have assessed the relationship between flare and CECD loss within the same cohort. CECD loss after cataract surgery varies widely among reports [13,14,15], and recent evidence suggests that controlling early postoperative inflammation may help reduce corneal endothelial cell loss and support long-term endothelial stability [16].

Corneal endothelial cells lack proliferative capacity and do not regenerate. Consequently, corneal endothelial cell loss triggers compensatory mechanisms such as cell enlargement, migration, and increased polymegathism, ultimately causing corneal endothelial heterogeneity [17]. When CECD falls below approximately 400 cells/mm^2^, corneal endothelial pump failure occurs, resulting in stromal over-hydration, corneal edema, bullous keratopathy, scarring, and irreversible visual impairment [18,19]. Pseudophakic bullous keratopathy is a serious complication of cataract surgery, and in the United States, 8.5% of corneal transplantation cases are attributable to cataract surgery [20]. Therefore, minimizing postoperative corneal endothelial cell loss remains a critical objective in modern phacoemulsification.

The Eight-chop technique aims to enhance the efficiency of phacoemulsification by mechanically dividing the nucleus into eight small, uniform segments [11]. This approach reduces the required ultrasound energy, shortens aspiration time, and minimizes intraocular manipulation. These features suggest that the Eight-chop technique may attenuate postoperative inflammation and thereby help preserve corneal endothelial integrity. Despite its theoretical advantages, no prior study has systematically evaluated postoperative flare and CECD loss in eyes undergoing the Eight-chop technique. Although postoperative inflammation has been described extensively in previous reports, studies that incorporate eye-level clustering using linear mixed-effects models (LMMs) are only sporadically found in the cataract literature. More importantly, to our knowledge, no published study has directly quantified the relationship between postoperative flare and CECD while appropriately accounting for within-patient inter-eye correlation using a mixed-effects modeling framework [21].

Therefore, the purpose of this study was to quantify postoperative changes in aqueous flare and CECD in eyes undergoing phacoemulsification with the Eight-chop technique; to determine whether early postoperative flare is associated with subsequent corneal endothelial cell loss; and to evaluate the relationships among flare, CECD loss, and intraoperative parameters. This study aims to provide new insights into postoperative inflammatory responses and corneal endothelial cell behavior following the Eight-chop technique and to contribute to a better understanding of minimally invasive cataract surgery.

## 2. Materials and Methods

### 2.1. Ethical Considerations

The research protocol was reviewed and approved by the institutional ethics committee and conducted in accordance with the tenets of the Declaration of Helsinki. The purpose of the study, including the use of clinical data for research, was explained to all patients prior to surgery, and written informed consent was obtained (approval number: 20250106).

### 2.2. Study Population

This prospective observational study included 70 patients (118 eyes) who underwent phacoemulsification using the Eight-chop technique at Sato Eye Clinic (Matsudo City, Japan) between 20 January 2025 and June 2025. Only non-diabetic patients were included in order to eliminate systemic inflammatory influence on postoperative flare and corneal endothelial evaluation. Exclusion criteria were as follows: diabetes mellitus; corneal disease or opacity; uveitis or glaucoma; retinal pathology such as diabetic retinopathy; congenital anomalies including microcornea, nanophthalmos, or anterior segment dysgenesis; history of ocular trauma or intraocular surgery; lens nucleus Emery-Little Grade IV or higher [22]; cases requiring iris retractors or capsular tension devices; intraoperative complications; and inability to maintain follow-up to 19 weeks. For bilateral cases, both eyes were included, and intra-patient correlation was accounted for in the statistical analysis using linear mixed-effects modeling.

### 2.3. Preoperative Assessment

All patients underwent comprehensive ocular examinations before surgery. Best-corrected visual acuity (BCVA) was measured using a decimal chart and converted to the logarithm of the minimum angle of resolution (logMAR). Intraocular pressure (IOP) was measured using a Goldmann applanation tonometer. Corneal endothelial parameters—including CECD, central corneal thickness (CCT), coefficient of variation (CV), and percentage of hexagonal cells (PHC)—were obtained using a non-contact specular microscope (EM-3000; Topcon Corporation, Tokyo, Japan). Anterior chamber depth (ACD) and axial length were measured with a sweep-source optical biometer using a 1060 nm wavelength (OA-2000; TOMEY, Tokyo, Japan).

### 2.4. Aqueous Flare Measurement

Aqueous flare was measured using a standardized laser flare photometer (Kowa FM-600, Kowa Company Ltd., Tokyo, Japan). Measurements were performed under controlled lighting conditions, and three consecutive scans were obtained and averaged. Flare intensity was expressed in photon counts per millisecond (photon counts/ms), the conventional output unit of laser flare photometry, which quantifies the amount of scattered photons in the anterior chamber and indicates the degree of postoperative intraocular inflammation.

### 2.5. Surgical Technique

All surgeries were performed by a single experienced cataract surgeon (T.S.), who is highly skilled in the Eight-chop technique. A 3.0 mm clear corneal incision was created using a steel keratome, after which sodium hyaluronate was injected into the anterior chamber. A continuous curvilinear capsulorrhexis measuring approximately 6.0–6.2 mm was created using capsulorrhexis forceps (F-2055; M.E. Technica, Tokyo, Japan). Hydrodissection was performed using a 27-gauge cannula (AMO Japan, Inc., Tokyo, Japan). The lens nucleus was divided into eight segments using an Eight-chopper II. Each segment was emulsified and aspiration using the Centurion^®^ Vision System (Alcon Laboratories, Inc., Irvine, CA, USA). Residual cortex was removed via irrigation/aspiration, and a foldable hydrophobic acrylic intraocular lens (Acrysof^®^ MN60AC; Alcon Laboratories, Inc., Fort Worth, TX, USA) was implanted into the capsular bag. Viscoelastic was thoroughly removed, and the anterior chamber was replenished with balanced salt solution containing moxifloxacin (0.5 mg/mL). The following intraoperative parameters were automatically recorded: phaco time (s), aspiration time (s), cumulative dissipated energy (CDE), and total irrigation fluid volume (mL). In contrast, the operative time (min) was manually measured from the initiation of the corneal incision to the completion of intraocular viscoelastic aspiration. All surgeries were recorded using a high-definition camera system (MKC-704KHD; Ikegami Tsushinki Co., Ltd., Tokyo, Japan) and stored digitally.

### 2.6. Postoperative Examinations

Postoperative evaluations were performed at Day 1, Day 7, Week 7, and Week 19. At each visit, BCVA, IOP, aqueous flare, CECD, CCT, CV, and PHC were measured using the same devices and protocols as preoperative assessments. Aqueous flare values were obtained in triplicate and averaged. Corneal endothelial cell density loss (CECD loss, %) was calculated using the following formula:

CECD loss (%) = (Preoperative CECD − Postoperative CECD)/Preoperative CECD × 100(1)

Similarly, IOP reduction (IOP reduction, %) was calculated as:

IOP reduction (%) = (Preoperative IOP − Postoperative IOP)/Preoperative IOP × 100(2)

In eyes where the postoperative CECD exceeded the preoperative value, we assumed that this apparent increase did not represent true corneal endothelial cell regeneration. Corneal endothelial cells are generally considered non-proliferative in vivo, and chronic corneal endothelial dysfunction is characterized by progressive cell loss rather than gain [23]. In addition, non-contact specular microscopy is known to exhibit a test–retest variability of several percent in CECD measurements [24]. Therefore, postoperative CECD values higher than baseline were interpreted as measurement noise rather than biological improvement. For statistical analysis, the percentage CECD loss in such eyes was set to 0%, and these eyes were retained in the dataset to avoid overestimation of corneal endothelial damage and to minimize selection bias.

### 2.7. Sample Size Considerations

This study was designed as a prospective observational study, and no formal a priori sample size calculation was performed. The sample size was determined based on the availability of eligible consecutive cases during the study period. Previous studies evaluating postoperative aqueous flare and CECD after phacoemulsification have reported comparable sample sizes (typically 50–120 eyes), which have been sufficient to detect clinically meaningful differences in early inflammatory response and corneal endothelial cell changes. Therefore, the present sample size was deemed adequate to ensure reliable estimation of postoperative flare and corneal endothelial outcomes and to perform linear mixed-effects modeling with appropriate statistical power.

### 2.8. Statistical Analysis

All statistical analyses were performed using R software (version 4.3.2; R Foundation for Statistical Computing, Vienna, Austria) with the lme4, lmerTest, dplyr, and readxl packages.

#### 2.8.1. Normality Assessment

The distribution of continuous variables (flare, BCVA, CCT, CV, PHC, and IOP) was assessed using visual inspection of histograms and Q–Q plots, as well as the Shapiro–Wilk test when necessary. Variables that approximated a normal distribution were analyzed using parametric methods, whereas non-normally distributed variables were compared using nonparametric alternatives.

#### 2.8.2. Comparison of Preoperative and Postoperative Measurements

Changes from baseline to postoperative time points (Day 1, Day 7, Week 7, and Week 19) were analyzed using paired *t*-tests for normally distributed variables (BCVA, CECD, CCT, CV, PHC, and IOP). For aqueous flare measurements, data distributions at each postoperative time point were considered potentially non-normal. Therefore, paired comparisons between preoperative and postoperative flare values were performed using the Wilcoxon signed-rank test.

#### 2.8.3. Linear Mixed-Effects Models for CECD Loss and IOP Reduction

To evaluate predictors of corneal endothelial cell loss and postoperative IOP reduction, LMMs were constructed using the lme4 package. Patient ID was included as a random intercept to account for within-subject correlation arising from bilateral surgeries. Because each patient contributed one or both eyes to the dataset, the observations were not statistically independent. Therefore, LMMs were used instead of ordinary linear regression to appropriately account for the hierarchical data structure. LMMs incorporate both fixed effects, representing population-level associations, and random effects, which model subject-specific variability. A random-intercept structure was adopted so that each patient had an individualized baseline level of flare or corneal endothelial cell loss. This approach corrects for the inherent similarity between paired eyes and prevents underestimation of standard errors that could occur if intra-eye correlation were ignored. Model parameters were estimated using restricted maximum likelihood, and statistical significance was evaluated using t-values based on Satterthwaite’s approximation. This modeling framework enabled a robust assessment of surgical predictors while properly adjusting for clustering within patients. Fixed effects included Day 1 flare, age, preoperative CECD, Emery-Little grade, CDE, and irrigation fluid volume for CECD loss models, and Day 1 flare for IOP reduction models. Model estimates (β coefficients), standard errors, and *p*-values were obtained via the lmerTest package. Linear mixed-effects modeling was performed using the lme4 package [25], and statistical inference was obtained using the lmerTest package [26]. For the linear mixed-effects models, regression coefficients are reported with 95% confidence intervals calculated using the Wald method.

#### 2.8.4. Multicollinearity Assessment

Multicollinearity among fixed predictors was evaluated using variance inflation factors (VIFs). All VIF values were <3, indicating the absence of problematic collinearity among explanatory variables.

#### 2.8.5. Statistical Significance

All tests were two-sided, and *p* < 0.05 was considered statistically significant. Descriptive statistics are presented as mean ± standard deviation. No formal adjustment for multiple comparisons was applied.

## 3. Results

### 3.1. Baseline Characteristics and Intraoperative Parameters

A total of 118 eyes from 70 patients were included in the analysis. Baseline and intraoperative characteristics are summarized in Table 1. The mean age was 73.4 ± 6.8 years, and 38 eyes (32.2%) were from male patients. Preoperative CECD averaged 2657.9 ± 191.0 cells/mm^2^, and the mean preoperative aqueous flare was 7.63 ± 2.52 (photon counts/ms). The mean operative time was 5.24 ± 1.05 min, with a mean phaco time of 17.1 ± 6.86 s and mean CDE of 6.13 ± 2.09. Mean irrigation fluid usage was 29.1 ± 9.23 mL.

### 3.2. Postoperative Aqueous Flare Dynamics

Postoperative flare values increased significantly at all postoperative time points compared with baseline (Table 2). Flare peaked at Day 7 (16.7 ± 9.21 photon counts/ms) and gradually decreased thereafter, reaching 10.5 ± 3.67 photon counts/ms at Week 19. All postoperative time points showed statistically significant increases relative to baseline (all *p* < 0.001).

### 3.3. Relationship Between Intraoperative Parameters and Day 1 Aqueous Flare

To determine whether postoperative inflammation was influenced by intraoperative surgical stress, a linear mixed-effects model was constructed using Day 1 aqueous flare as the dependent variable and intraoperative parameters as fixed effects (Table 3). None of the evaluated intraoperative variables—including CDE, irrigation fluid volume, operative time, phaco time, or aspiration time—showed a significant association with postoperative Day 1 flare (all *p* > 0.30). Emery-Little nuclear grade was also not a significant predictor (*p* = 0.394). The random intercept for Patient ID indicated modest between-subject variability (variance = 15.72), whereas the residual variance remained larger (26.75), suggesting that differences in flare responses were largely unrelated to intraoperative factors.

### 3.4. Changes in Corneal Endothelial Cell Density

Changes in CECD are summarized in Table 4. Mean CECD decreased slightly from 2657.9 ± 191.0 cells/mm^2^ preoperatively to: 2621.2 ± 188.3 cells/mm^2^ at Week 7, 2619.0 ± 198.0 cells/mm^2^ at Week 19. Corresponding corneal endothelial cell loss rates were: 1.38% at Week 7, 1.46% at Week 19. Both reductions were statistically significant (*p* < 0.001) but clinically very small.

### 3.5. Linear Mixed-Effects Models for CECD Loss

#### 3.5.1. Predictors of CECD Loss at Week 7

The linear mixed-effects model identified Emery grade (β = 1.67, *p* = 0.004) and preoperative CECD (β = 0.00219, *p* = 0.028) as significant predictors of Week 7 CECD loss. Day 1 flare was not a significant predictor (β = −0.028, *p* = 0.35) (Table 5).

#### 3.5.2. Predictors of CECD Loss at Week 19

At Week 19, only Emery grade (β = 1.40, *p* = 0.025) remained a significant positive predictor. Again, Day 1 flare showed no association with CECD loss (β = −0.0057, *p* = 0.85). These results indicate that lens hardness and surgical energy influence CECD loss, but postoperative inflammatory response does not (Table 6).

In the linear mixed-effects model evaluating percentage corneal CECD change at postoperative week 19, greater irrigation fluid volume was associated with a statistically significant increase in CECD loss (estimate: 0.083% per mL; 95% confidence interval [CI], 0.008 to 0.157) (Table 7). Emery-Little nuclear grade showed a borderline association with CECD loss (estimate: 1.15% per grade; 95% CI, 0.02 to 2.29). Age, preoperative CECD, and phacoemulsification time were not significantly associated with CECD loss, and their confidence intervals included zero.

Values are presented as regression coefficients with corresponding 95% confidence intervals (CI) derived using the Wald method. The linear mixed-effects model included Emery-Little nuclear grade, patient age, preoperative CECD, phacoemulsification time, and total irrigation fluid volume as fixed effects, with patient ID specified as a random intercept to account for inter-eye correlation. The marginal and conditional R^2^ values for the model were 0.093 and 0.258, respectively.

### 3.6. Postoperative Intraocular Pressure

Mean IOP decreased significantly after surgery:Preoperative: 13.8 ± 1.84 mmHg, Week 7: 12.2 ± 2.06 mmHg, Week 19: 12.3 ± 2.12 mmHg. Paired *t*-tests showed significant reductions at both Week 7 and Week 19 (both *p* < 0.001). Linear mixed models demonstrated no significant correlation between IOP reduction and Day 1 flare at either time point (Table 8).

### 3.7. Corneal Morphological Parameters

CCT showed only minimal changes (Table 9): Slight increase at Week 7 (*p* = 0.002), No significant change by Week 19 (*p* = 0.12). CV remained stable at Week 7 but decreased significantly at Week 19 (*p* < 0.001) (Table 9). This suggests improvement in cell size regularity over time. PHC remained stable at Week 7 but increased significantly at Week 19 (*p* = 0.004) (Table 8). Together, CV and PHC changes imply that morphological recovery of the endothelium continues beyond 7 weeks.

### 3.8. Best-Corrected Visual Acuity

BCVA improved significantly after surgery (Table 10): Preoperative: 0.098 ± 0.180 logMAR, Week 7: −0.064 ± 0.035 logMAR, Week 19: −0.066 ± 0.030 logMAR. Improvements at both postoperative time points were statistically significant (*p* < 0.001).

### 3.9. Complications

No intraoperative complications were encountered in any case. Specifically, there were no capsulorhexis tears, posterior capsule ruptures, zonular dialysis, or iris trauma during surgery. Postoperatively, no cases of persistent corneal edema, anterior chamber fibrin reaction, or endophthalmitis were observed throughout the 19-week follow-up period. These findings indicate that the Eight-chop technique can be performed safely and reproducibly, with a very low incidence of intra- and postoperative complications.

## 4. Discussion

Cataract surgery performed using the Eight-chop technique demonstrated a characteristic postoperative inflammatory profile and exceptionally small corneal endothelial cell loss in this prospective observational study. The present findings provide comprehensive insight into the intraocular response after Eight-chop phacoemulsification and clarify the relationships among postoperative aqueous flare, intraoperative surgical parameters, and corneal endothelial preservation. To our knowledge, this is the first study to evaluate early flare, CECD, morphological corneal endothelial parameters, and IOP changes simultaneously using a unified surgical technique, with all operations performed by a single surgeon experienced in the Eight-chop method. This study provides important evidence regarding the low-invasive nature of this technique and its potential to minimize intraoperative and postoperative ocular tissue stress.

Early postoperative flare is widely recognized as an indicator of acute inflammatory response following phacoemulsification [27,28]. Previous studies consistently reported Day 1 flare values between approximately 27 and 31 photon counts/ms, including classical parameters such as 30.6 ± 15.7 and 27.8 ± 4.4 photon counts/ms, depending on surgical technique and patient-specific variables [27,28]. These values have been regarded as the typical magnitude of postoperative breakdown of the blood–aqueous barrier in conventional ultrasound-dependent phacoemulsification. In contrast, the present study observed a substantially lower Day 1 flare level of 15.1 photon counts/ms, far below the previously established ranges. This clearly indicates that the Eight-chop technique attenuates the inflammatory response in the immediate postoperative period. Although flare increased on postoperative Day 1 and Day 7 as expected after cataract extraction, the magnitude of elevation was modest and diminished rapidly by Week 7, with continued improvement through Week 19. This clinical pattern suggests that the Eight-chop technique minimizes mechanical contact, fluid turbulence, and ultrasound exposure within the anterior chamber.

In evaluating the mechanistic foundation of this minimal flare response, the principled characteristics of the Eight-chop technique must be considered. In conventional phaco-chop or divide-and-conquer techniques, ultrasound energy is applied from the very beginning of nuclear division. In contrast, the Eight-chop technique performs complete mechanical fragmentation of the nucleus before phacoemulsification, dividing it into eight small and uniformly shaped segments. This mechanical disassembly markedly reduces the amount of ultrasound energy required during emulsification and greatly improves aspiration efficiency, thereby shortening operative time, aspiration duration, and irrigation fluid usage. Reductions in aspiration time and irrigation volume are particularly important, as fluctuations in inflow and outflow are known to increase shear stress on the corneal endothelium and iris, which can elevate early postoperative flare [29]. By suppressing these fluidic disturbances, the Eight-chop technique establishes a less invasive intraocular environment and minimizes both mechanical and thermal trauma to uveal tissues [30].

A central finding of this study was the complete lack of association between Day 1 flare and any intraoperative parameter, including CDE, irrigation fluid volume, operative time, phaco time, aspiration time, or lens hardness. Linear mixed-effects modeling revealed that none of these conventional surgical markers significantly predicted postoperative flare intensity. The regression coefficients were small and nonsignificant across all variables, indicating that postoperative inflammation remained uniformly low regardless of nuclear hardness or intraoperative energy delivery. Moreover, the residual variance in the model was approximately 26.75, substantially exceeding the random-effect variance attributed to patient-specific factors (15.72), suggesting that most of the variation in flare was not explained by intraoperative differences. This pattern strongly supports the conclusion that the Eight-chop technique standardizes the surgical stress applied to intraocular tissues, resulting in consistently low inflammatory responses across cases.

This homogeneity in postoperative flare is highly unusual in phacoemulsification literature. Numerous studies have reported that higher ultrasound energy, longer operative time, or larger fluid volumes are associated with elevated postoperative flare [31,32,33]. For example, prior analyses demonstrated that cataracts requiring prolonged ultrasound exposure generated higher levels of early flare due to increased blood–aqueous barrier breakdown [34]. However, the present findings suggest that the Eight-chop technique suppresses the pathway through which ultrasonic and fluidic stress translates into inflammatory response. These results align with the mechanistic advantages previously proposed in earlier Eight-chop research, including reduced fragmentation time, minimized turbulence, and rapid aspiration of pre-segmented nuclear fragments. The lack of correlation between Day 1 flare and surgical parameters underscores the consistent, low-trauma performance of the technique, providing important evidence that Eight-chop may represent a less invasive alternative to standard phaco-chop or divide-and-conquer.

The extremely small corneal endothelial cell loss observed in this study further supports the minimally invasive nature of the Eight-chop technique. Mean CECD loss was only 1.38% at Week 7 and 1.46% at Week 19, far lower than previously reported values of 4.4–18.8% following standard phacoemulsification techniques [35,36]. These values remained stable over time, indicating that early corneal endothelial cell loss was minimal and did not progress. The magnitude of corneal endothelial preservation observed herein is clinically meaningful, especially in elderly patients or those with borderline corneal endothelial reserves. The linear mixed-effects model identified lens hardness (Emery-Little grade) as the only consistent predictor of corneal endothelial cell loss, in agreement with existing literature that associates nuclear density with greater intraoperative trauma. CDE and fluid volume showed borderline associations with cell loss, reflecting the influence of energy delivery but also the limited magnitude of that effect within the Eight-chop framework. Although several factors showed statistically significant associations with postoperative CECD change, the estimated effect sizes were small, and their 95% confidence intervals in some cases overlapped with the known test–retest variability of non-contact specular microscopy; therefore, these findings should be interpreted cautiously, with emphasis on statistical uncertainty and limited clinical magnitude rather than on *p* values alone.

The lack of association between postoperative flare and corneal endothelial cell loss is notable. In more invasive phacoemulsification approaches, postoperative inflammation has been implicated in corneal endothelial damage through cytokine-mediated mechanisms, including oxidative stress and nitric oxide–related injury [37]. However, in the present cohort, flare levels were so low and uniform that they failed to predict corneal endothelial decline. This dissociation suggests that corneal endothelial injury during Eight-chop is determined more by direct surgical factors—such as lens hardness and intraoperative ultrasound energy—than by postoperative inflammatory activity. The Eight-chop technique’s minimal ultrasound usage and stable fluidics likely reduce direct shear and mechanical insult, thereby preventing incremental corneal endothelial damage independent of flare activity. This finding has important clinical implications: flare measurement may have limited value in predicting corneal endothelial outcomes when the surgical technique is inherently less invasive.

Corneal morphological indices, including CCT, CV, and PHC, provided further quantitative evidence of corneal endothelial preservation. CCT showed only a small transient increase at Week 7, returning toward baseline at Week 19. Moreover, significant improvements in CV and PHC at Week 19 reflected stabilization and recovery of corneal endothelial cell morphology. These morphological improvements contrast with the patterns seen after more invasive phacoemulsification techniques, where sustained increases in CV and decreases in PHC often reflect ongoing corneal endothelial stress or delayed healing [38,39]. The normalization of corneal endothelial morphology in this study reinforces the concept that the Eight-chop technique imposes minimal long-term biomechanical stress.

Visual acuity recovery was rapid, with dramatic improvements in BCVA by Week 7 and further gains at Week 19. This early recovery is consistent with minimal corneal edema, limited tissue trauma, and efficient removal of nuclear fragments without excessive manipulation. Intraocular pressure (IOP) exhibited a significant and sustained postoperative reduction, averaging 12–13% at Week 7 and Week 19. Although cataract surgery is known to lower IOP through enhanced aqueous outflow [40,41], the Eight-chop technique may provide an additional advantage by minimizing fluidic stress on the trabecular meshwork and Schlemm’s canal. Linear mixed-effects modeling showed no association between IOP reduction and postoperative flare, confirming that early inflammation does not compromise the outflow pathway in the context of Eight-chop’s low-invasive approach.

The clinical implications of these findings are substantial. Eight-chop appears to offer a highly reproducible surgical method that standardizes intraocular stress, minimizes reliance on ultrasound energy, and reduces postoperative inflammation across a wide range of cataract densities. Surgeons managing patients with fragile corneal endothelium—such as elderly individuals, patients with Fuchs endothelial dystrophy, shallow anterior chambers, or pre-existing borderline cell counts—may benefit from adopting this technique. Given its low fluid requirements and gentle manipulation of nuclear fragments, the Eight-chop technique could serve as a viable alternative to femtosecond laser-assisted cataract surgery [42,43,44], particularly when cost or accessibility is limiting. Furthermore, the minimal corneal endothelial damage associated with Eight-chop may lower the risk of long-term complications such as persistent corneal edema, bullous keratopathy, or delayed corneal endothelial decompensation.

Recent independent literature has also acknowledged the potential advantages of the Eight-chop technique. A 2025 international review on cataract surgery in diabetic eyes cited preliminary clinical studies of the Eight-chop method and described it as a low-invasiveness technique that reduces ultrasound dependence, minimizes fluidic stress, and may help preserve corneal endothelial function in metabolically vulnerable eyes [45]. These external evaluations are consistent with the mechanistic and clinical findings of the present study and further support the position of Eight-chop as a promising refinement of modern phacoemulsification.

To minimize confounding in the assessment of postoperative inflammation, patients with diabetes were excluded from this study. Therefore, the present findings represent the performance of the Eight-chop technique in a non-diabetic population, and its effects in diabetic patients cannot be determined from these data. This study evaluated the clinical performance of the Eight-chop technique alone and did not include direct comparisons with other cataract surgery methods. Therefore, randomized controlled trials comparing Eight-chop phacoemulsification with other techniques are required to fully assess its relative advantages in terms of safety, operability, and cost-effectiveness. This study does have limitations, including the absence of a direct comparison group using phaco-chop, divide-and-conquer, or Phaco Pre-chop techniques. However, extensive prior literature describing typical flare patterns and corneal endothelial outcomes allows the present results to be contextualized appropriately. The observation period was limited to 19 weeks, and longer follow-up would be required to determine whether subtle corneal endothelial changes emerge beyond this timeframe. Additionally, although the linear mixed-effects model accounted for inter-eye correlation, potential confounding factors not captured in the dataset may influence inflammatory or corneal endothelial responses. Because bilateral cases were included, patient ID was specified as a random effect to account for inter-eye correlation. However, the potential influence of first-eye surgery on the baseline condition or postoperative inflammatory response of the fellow eye cannot be excluded and represents a limitation of this study. Future studies should consider surgical order effects or restrict analyses to unilateral cases.

## 5. Conclusions

Eight-chop phacoemulsification was associated with low levels of early postoperative intraocular inflammation and a small magnitude of corneal endothelial cell loss. Aqueous flare on postoperative day 1 was lower than values typically reported after conventional phacoemulsification and showed no significant association with intraoperative parameters, suggesting a consistently low degree of intraocular tissue stress with this technique. Although the observed changes in corneal endothelial cell density were within the expected measurement variability, favorable endothelial preservation and subsequent morphological recovery were observed over time. Taken together, these findings indicate that the Eight-chop technique is an efficient and low-invasive surgical approach that may reduce intraocular stress and offer potential advantages over conventional ultrasound-dependent phacoemulsification, while acknowledging the limitations inherent in endothelial cell measurements.

## Figures and Tables

**Table 1:** Preoperative characteristics and intraoperative parameters.

Variable	Mean ± SD or n (%)
Age (years)	73.4 ± 6.8
Sex (male/female)	38 (32.2%)/80 (67.8%)
Laterality (right/left eye)	57 (48.3%)/61 (51.7%)
Best-corrected visual acuity (logMAR)	0.098 ± 0.180
Anterior chamber depth (mm)	3.20 ± 0.39
Axial length (mm)	23.98 ± 1.66
Intraocular pressure (mmHg)	13.82 ± 1.84
Corneal endothelial cell density (cells/mm^2^)	2658 ± 191
Central corneal thicness (µm)	527.0 ± 35.2
Coefficient of variation in cell area (%)	39.20 ± 5.31
Percentage of hexagonal cells (%)	45.26 ± 7.09
Lens nuclear hardness (Emery-Little grade)	2.36 ± 0.36
Operative time (min)	5.24 ± 1.05
Phaco time (s)	17.11 ± 6.86
Aspiration time (s)	78.15 ± 20.75
Cumulative dissipated energy	6.13 ± 2.09
Irrigation fluid volume (mL)	29.07 ± 9.23

**Table 2:** Aqueous flare at each postoperative time point.

Time Point	Mean ± SD (Photon Units/ms)	*p*-Value vs. Preoperative
Preoperative	7.63 ± 2.52	—
Postoperative Day 1	15.10 ± 6.36	*p* < 0.001
Postoperative Day 7	16.70 ± 9.21	*p* < 0.001
Week 7	12.90 ± 6.47	*p* < 0.001
Week 19	10.50 ± 3.67	*p* < 0.001

**Table 3:** Linear mixed-effects model for predictors of postoperative Day 1 aqueous flare.

Variable	Estimate	Standard Error	*p*-Value
Operative time (min)	0.344	0.915	0.708
Phaco time (s)	−0.032	0.216	0.881
Aspiration time (s)	−0.055	0.088	0.531
Cumulative dissipated energy	0.309	0.508	0.545
Irrigation fluid volume (mL)	0.208	0.219	0.345
Emery-Little grade	−1.780	2.075	0.394

Random effect: Patient ID (variance = 15.72). Model residual variance = 26.75.

**Table 4:** Corneal endothelial cell density and percentage loss.

Time Point	CECD (Cells/mm^2^) Mean ± SD	CECD Loss (%)	*p*-Value vs. Preoperative
Preoperative	2665 ± 191		
Week 7	2638 ± 194	0.99%	*p* < 0.001
Week 19	2644 ± 198	0.77%	*p* < 0.001

**Table 5:** Linear mixed-effects model for CECD loss at Week 7.

Variable	Estimate	Standard Error	t-Value	*p*-Value
Intercept	−10.816	4.425	−2.44	0.017
Day 1 flare	−0.035	0.036	−0.95	0.343
Age (years)	0.031	0.037	0.83	0.408
Preoperative CECD	0.002	0.0017	1.72	0.089
Emery-Little grade	1.764	0.697	2.53	0.014
Cumulative dissipated energy	−0.218	0.149	−1.46	0.147
Irrigation fluid volume	0.060	0.030	2.00	0.049

Random effect: Patient ID (variance = 0.144).

**Table 6:** Linear mixed-effects model for CECD loss at Week 19.

Variable	Estimate	Standard Error	t-Value	*p*-Value
Intercept	−9.834	5.290	−1.86	0.067
Day 1 flare	−0.047	0.041	−1.14	0.259
Age (years)	0.092	0.045	2.07	0.042
Preoperative CECD	0.0004	0.001	0.31	0.757
Emery-Little grade	1.202	0.838	1.43	0.156
Cumulative dissipated energy	−0.266	0.170	−1.57	0.119
Irrigation fluid volume	0.077	0.033	2.31	0.023

Random effect: Patient ID (variance = 1.872).

**Table 7:** Linear mixed-effects model results showing fixed-effect coefficients and 95% confidence intervals for percentage CECD change at postoperative week 19.

Predictor	Estimate	95% CI (Lower)	95% CI (Upper)	*p*-Value
Emery-Little grade	1.153	0.016	2.290	0.051
Age (years)	0.053	−0.009	0.116	0.097
Preoperative CECD	0.0004	−0.002	0.003	0.692
Phaco time (s)	−0.104	−0.209	0.002	0.057
Fluid volume (mL)	0.083	0.008	0.157	0.032

**Table 8:** Linear mixed-effects models for postoperative IOP reduction (Week 7 and Week 19).

Model	Variable	Estimate	Standard Error	t-Value	*p*-Value
Week 7 IOP reduction	Intercept	8.557	2.397	3.570	<0.001
	Day 1 flare	0.237	0.133	1.780	0.078
Week 19 IOP reduction	Intercept	11.427	2.684	4.257	<0.001
	Day 1 flare	−0.004	0.149	−0.030	0.976

Random effect: Patient ID variance = 89.4 (Week 7)/113.7 (Week 19).

**Table 9:** Changes in corneal morphological parameters (CCT, CV, PHC) before and after cataract surgery.

Parameter	Time Point	Mean ± SD	*p*-Value vs. Preoperative
Central corneal thickness (µm)	Preoperative	527.0 ± 35.2	—
	Week 7	531.0 ± 35.1	*p* < 0.01
	Week 19	529.0 ± 35.3	*p* = 0.12
Coefficient of variation in corneal endothelial cell area (%)	Preoperative	39.20 ± 5.31	—
	Week 7	39.40 ± 5.88	*p* = 0.576
	Week 19	37.30 ± 4.39	*p* < 0.001
Percentage of hexagonal corneal endothelial cells (%)	Preoperative	45.26 ± 7.09	—
	Week 7	45.20 ± 6.92	*p* = 0.953
	Week 19	46.80 ± 5.81	*p* < 0.01

**Table 10:** Best-corrected visual acuity.

Time Point	Mean logMAR ± SD	*p*-Value vs. Preoperative
Preoperative	0.098 ± 0.180	—
Week 7	−0.064 ± 0.035	*p* < 0.001
Week 19	−0.066 ± 0.030	*p* < 0.001

## Data Availability

The anonymized datasets generated and analyzed during the present study are available in the Supplementary Materials (Supplementary Dataset S1 and S2). No personally identifiable information is included, in accordance with ICMJE data sharing standards.

## Supplementary Materials

The following supporting information can be downloaded at: https://www.mdpi.com/article/10.3390/jcm15020557/s1, Supplementary Dataset S1: Minimal_EightChop; Supplementary Dataset S2: Full_EightChop.

## Abbreviations

The following abbreviations are used in this manuscript:

CECD	Corneal endothelial cell density
LMMs	Linear mixed-effects models
CDE	Cumulative dissipated energy
BCVA	Best-corrected visual acuity
logMAR	Logarithm of the minimum angle of resolution
IOP	Intraocular pressure
CCT	Central corneal thickness
CV	Coefficient of variation
PHC	Percentage of hexagonal cells
